# Lentiviral hTERT Overexpression Extends the Lifespan of Primary Human Epidermal Melanocytes and Reshapes Transcriptional Programs

**DOI:** 10.3390/biom16091239

**Published:** 2026-08-27

**Authors:** Qiaohua Li, Shaoxuan Liu, Jingxing Gao, Jie Li, Hong Shi

**Affiliations:** State Key Laboratory of Primate Biomedical Research, Institute of Primate Translational Medicine, Kunming University of Science and Technology, Kunming 650500, China; liqiaohua@stu.kust.edu.cn (Q.L.); liushaoxuan@stu.cpu.edu.cn (S.L.); gaojingxing@stu.kust.edu.cn (J.G.); lij@stu.kust.edu.cn (J.L.)

**Keywords:** primary epidermal melanocyte, hTERT, lentivirus, lifespan extension, RNA-seq, pigmentation, cell model

## Abstract

Primary human epidermal melanocytes are valuable models for pigmentation research, but maintaining prolonged expansion together with stable melanocytic characteristics can be challenging. Here, primary melanocytes were transduced with an hTERT-overexpression lentiviral vector to generate a mixed population (MIX) and three single-cell-derived clones (SC1, SC3, and SC4). RNA sequencing was performed on passage-6 primary melanocytes, MIX cells, SC clones, and three melanoma cell lines (A375, WM-115, and SK-MEL-1). hTERT overexpression extended melanocyte culture lifespan by more than 10 passages. TERT expression increased in MIX cells and was highest in SC clones. Principal component and gene-expression analyses showed that MIX and SC cells remained globally closer to primary melanocytes than to melanoma cell lines, without an evident melanoma-like transcriptomic shift. However, lifespan extension and clonal selection were accompanied by marked morphological changes and broad transcriptional remodeling involving cell-cycle regulation, telomere-associated processes, antiviral responses, extracellular matrix organization, adhesion, dendrite-related programs, and pigmentation. Melanocyte differentiation and pigmentation programs were generally reduced, particularly in clonal cultures. These findings support hTERT-extended melanocytes as expandable in vitro models, while highlighting that hTERT expression and single-cell cloning can induce functional model drift.

## 1. Introduction

Human epidermal melanocytes are specialized pigment-producing cells located mainly in the basal layer of the epidermis [1,2]. They synthesize melanin within melanosomes and transfer mature melanosomes to neighboring keratinocytes, thereby contributing to skin color and protection against ultraviolet radiation-induced damage [3,4,5]. Because of these functions, primary human epidermal melanocytes provide a biologically relevant model for studies of melanogenesis, ultraviolet responses, pigmentary disorders, melanocyte differentiation, and the early cellular context from which melanoma may arise [6,7,8].

Despite their physiological relevance, primary melanocytes can present practical challenges in long-term experiments. Their proliferative capacity in vitro is strongly influenced by culture conditions and mitogenic support. Previous studies have shown that factors such as basic fibroblast growth factor, insulin, and bovine pituitary extract contribute substantially to the proliferation of normal human epidermal melanocytes, and that withdrawal of specific growth supplements can markedly reduce their proliferative capacity [9]. Classical studies demonstrated that normal human epidermal melanocytes can undergo substantial proliferation and serial propagation under optimized culture conditions [10,11]. Nevertheless, maintaining prolonged expansion together with stable melanocytic characteristics remains an important consideration for experimental studies requiring repeated passaging, genetic manipulation, drug screening, or omics-based analyses.

Several strategies have been developed to overcome the limited lifespan of primary cells [12,13]. Viral oncogenes such as SV40 large T antigen or HPV E6/E7 can bypass p53/Rb-dependent checkpoints, but these approaches introduce strong oncogenic perturbations and may substantially alter cell identity [14,15]. By contrast, ectopic expression of hTERT is generally considered a milder strategy because it compensates for telomere shortening, one of the major drivers of replicative senescence [16,17]. For this reason, hTERT overexpression has often been used to establish lifespan-extended or immortalized primary cell models [18,19].

However, hTERT overexpression should not be assumed to be biologically inert [20,21,22,23]. Telomerase activation is closely related to proliferative capacity and is frequently observed in cancer [24,25]. In addition to maintaining telomeres, hTERT may influence gene expression, cell-cycle control, stress responses, differentiation state, and interactions with the extracellular matrix [26]. These effects are likely to be cell-type dependent. In melanocytes, which are highly differentiated and tightly linked to pigmentation, dendrite formation, and epithelial microenvironmental cues, enforced hTERT expression may generate consequences that differ from those observed in fibroblasts or epithelial cells.

Another critical issue is clonal selection. Commercial cell lines are frequently generated from single cells to achieve a stable and homogeneous cell population. However, single-cell cloning imposes intense selection pressure. Only cells with sufficient survival, stress tolerance, and proliferative capacity expand into clonal cultures. In primary melanocytes, this selection may preferentially enrich dedifferentiated cells or cells that have lost energetically costly melanocytic functions such as melanin synthesis [27,28,29]. Therefore, clonal hTERT-overexpressing melanocytes may not necessarily represent normal epidermal melanocytes more faithfully than bulk hTERT-overexpressing cultures.

In the present study, we evaluated the biological consequences of lentiviral hTERT overexpression in human epidermal melanocytes. We generated hTERT-overexpressing melanocyte cultures and established single-cell-derived cultures to mimic commercial cell-line construction. By comparing primary melanocytes, mixed hTERT-overexpressing melanocytes, single-cell-derived hTERT-overexpressing melanocytes, and representative melanoma cell lines using mRNA sequencing [30,31,32], we aimed to determine whether hTERT overexpression drives melanocytes toward a melanoma-like state, whether it promotes dedifferentiation, and whether clonal selection further modifies melanocyte identity.

## 2. Materials and Methods

### 2.1. Cell Culture and Cell Lines

**Figure 1:**
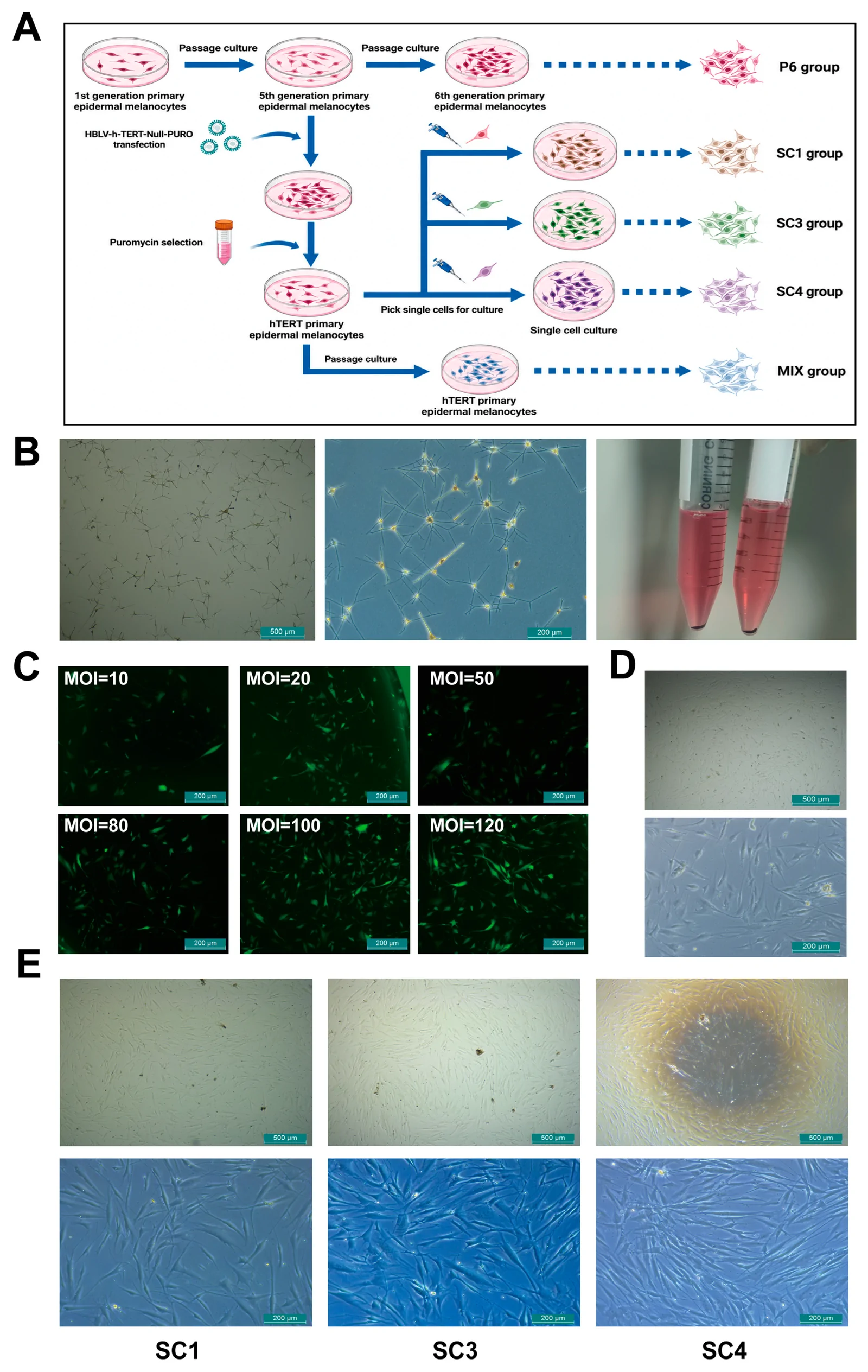
Establishment of hTERT-overexpressing primary human epidermal melanocyte models. (**A**) Experimental workflow. Primary epidermal melanocytes were passaged to P6 as the parental comparator. Fifth-passage cells were transduced with hTERT-overexpression lentivirus, selected with puromycin, and expanded either as a mixed hTERT-overexpressing population (MIX) or as single-cell-derived clonal lines (SC1, SC3, and SC4). Solid arrows indicate experimental procedures and cell-line derivation steps, whereas dotted arrows indicate assignment of the resulting cultures to the corresponding experimental groups used for downstream analyses. (**B**) Representative morphology of primary melanocytes. (**C**) Optimization of lentiviral infection using ZsGreen control virus across MOI values. (**D**) Representative morphology of mixed hTERT-overexpressing cultures. (**E**) Representative late morphology of SC1, SC3 and SC4 cultures.

Primary human epidermal melanocytes were purchased from Wuhan SAIOS Biotechnology Co., Ltd. (Wuhan, China; Cat. No. PC-088h) and were supplied as primary cells without prior in vitro passaging. After receipt and recovery, the initial culture established in our laboratory was designated passage 1 (P1), corresponding to the “1st generation” shown in Figure 1A. The cells were cultured in SAIOS primary melanocyte complete culture medium (Wuhan SAIOS Biotechnology Co., Ltd.; Cat. No. M-017) according to the manufacturer’s instructions. The complete culture system contained basal medium, melanocyte growth supplement, and fetal bovine serum at a final concentration of 0.5%. Cells were maintained at 37 °C in a humidified incubator with 5% CO_2_ and serially passaged at a ratio of 1:2. For routine passaging, adherent cells were detached using 0.25% trypsin-EDTA (Gibco, Thermo Fisher Scientific, Waltham, MA, USA; Cat. No. 25200056). After cell detachment, trypsin activity was terminated with complete melanocyte culture medium, and the cells were collected and reseeded in fresh medium. The cultures were serially expanded from P1 to P5. At P5, a portion of the culture was subjected to lentiviral hTERT transduction, whereas an untreated parallel culture was further passaged once to P6 and used as the parental comparator.

The melanoma cell lines A375, WM-115 and SK-MEL-1 were used as comparator models. A375 cells were cultured in complete DMEM containing 10% fetal bovine serum and 1% penicillin-streptomycin. WM-115 and SK-MEL-1 cells were cultured in complete MEM containing 10% fetal bovine serum and 1% penicillin-streptomycin. Cells were maintained at 37 °C in a humidified incubator with 5% CO_2_. Mycoplasma testing was performed before downstream experiments and repeated during cell-line establishment.

### 2.2. Optimization of Lentiviral Transduction

A ZsGreen-puromycin control lentivirus was used to optimize lentiviral infection conditions in primary epidermal melanocytes. Cells were seeded into 24-well plates at 1 × 10^4^ cells per well. After attachment, cultures were exposed to virus-containing medium across a multiplicity of infection (MOI) gradient of 0, 1, 2, 3, 5, 10, 20, 40, 50, 60, 80, 100, 120 and 150. A half-volume infection protocol was used: virus-containing medium was added for 4 h, after which fresh medium was added to restore the full culture volume. Medium was replaced approximately 24 h after infection. Fluorescence and cell morphology were evaluated 48–72 h after infection.

Polybrene toxicity was tested at 4 μg/mL. Under the conditions used here, Polybrene caused marked cytotoxicity in primary epidermal melanocytes and was therefore omitted from subsequent transductions. Puromycin sensitivity was assessed using 0, 0.5, 1.0, 1.5, 2.0, 4.0, 6.0, and 8.0 μg/mL puromycin for 48 h. The lowest concentration that killed more than 90% of cells was 1.0 μg/mL, which was selected for post-transduction enrichment.

### 2.3. Generation of hTERT-Overexpressing Melanocyte Populations and Single-Cell Clones

Fifth-passage primary melanocytes were transduced with an hTERT-overexpression lentiviral vector (HBLV-h-TERT-Null-PURO; transcript NM_198253.2; CDS length 3399 bp; delivered titer 2 × 10^8^ TU/mL). Three wells with healthy adherent cells were infected under the optimized conditions without Polybrene. At 72 h after infection, cells were selected with 1.0 μg/mL puromycin for 48 h and then returned to fresh melanocyte medium.

After selection, surviving hTERT-overexpressing cells were expanded as a mixed population (MIX). To mimic the clonal establishment procedure used for commercial cell lines, single cells were manually picked from the post-selection hTERT-overexpressing population into 96-well plates. Four clones were initially expanded and were named SC1, SC2, SC3, and SC4. SC2 stopped proliferating after transfer to 12-well culture, whereas SC1, SC3, and SC4 expanded to more than 1 × 10^6^ cells and were used for RNA-seq.

### 2.4. RNA Sequencing and Analysis

A375, WM-115, SK-MEL-1, untreated passage-6 primary melanocytes (P6), MIX, SC1, SC3, and SC4 cells were each prepared in triplicate. Before library preparation, adherent cells were detached using 0.25% trypsin-EDTA and collected as single-cell suspensions, whereas SK-MEL-1 suspension cells were collected directly. Each sample contained more than 1 × 10^5^ cells. Cells were washed with PBS, pelleted, snap-frozen in liquid nitrogen, and stored at −80 °C. Library construction and sequencing were performed by Novogene Co., Ltd. (Beijing, China) using low-input RNA-seq library preparation.

Sequencing reads were quality-checked using fastp (version 0.23.4) and aligned to the human GRCh38/hg38 reference genome with HISAT2 (version 2.2.1) [33,34]. Downstream analyses were performed in R version 4.3.1. Gene identifiers were converted using org.Hs.eg.db and clusterProfiler (version 4.10.0). Transcripts per million (TPM) values were calculated for expression visualization, and raw read counts were normalized for differential expression analysis. DESeq2 (version 1.42.0) was used to compare gene expression among groups [35]. Comparisons included P6 versus MIX, MIX versus SC, and P6 versus SC. Differentially expressed genes were visualized using volcano plots and heatmaps generated with ggplot2 (version 3.4.4), ggrepel (version 0.9.5), pheatmap (version 1.0.12), and ComplexHeatmap (version 2.18.0) [36]. For volcano-plot visualization, genes meeting adjusted *p* < 0.05 and absolute log2 fold change > 2 were highlighted. Tumor-related genes were curated from literature and database searches. P6, MIX, and SC samples were assigned to a non-tumor melanocyte model group, whereas A375, WM-115, and SK-MEL-1 samples were assigned to the melanoma comparator group. Tumor-related gene expression was visualized using ComplexHeatmap and circlize (version 0.4.16). Gene Ontology (GO) and Kyoto Encyclopedia of Genes and Genomes (KEGG) enrichment analyses were performed using clusterProfiler and org.Hs.eg.db. Gene set enrichment analysis (GSEA) was performed using fgsea (version 1.28.0), GSEABase (version 1.64.0), clusterProfiler, enrichplot (version 1.22.0), and msigdbr (version 7.5.1) with MSigDB gene sets [37].

## 3. Results

### 3.1. Lentiviral hTERT Overexpression Extended the Culture Lifespan of Primary Epidermal Melanocytes

The experimental workflow is shown in Figure 1A. Primary epidermal melanocytes were serially expanded in culture. At P5, a portion of the culture was transduced with the hTERT-overexpression lentiviral vector and selected with puromycin, after which the surviving cells were either expanded as a mixed population (MIX) or subjected to single-cell clonal expansion to generate SC lines. An untreated parallel culture was further passaged to P6 and used as the parental comparator. Primary melanocytes displayed the expected dendritic morphology during early culture (Figure 1B). Under the culture conditions used in the present study, the untreated primary melanocyte cultures showed a progressive decline in population expansion during continued serial passage. In the transduction-optimization experiment, Polybrene at 4 μg/mL caused marked cytotoxicity and was therefore omitted from subsequent transductions. Among the tested MOI values, an MOI of 100 provided the best balance between transduction efficiency and preservation of cell morphology, based on ZsGreen fluorescence and morphological assessment (Figure 1C). Puromycin at 1.0 μg/mL was sufficient for post-transduction selection.

Following hTERT transduction and puromycin selection, the mixed hTERT-overexpressing population continued to grow for more than 10 additional passages. During early culture after transduction, hTERT-overexpressing cells retained bipolar or multipolar dendritic morphology (Figure 1D), but at the late stage used for RNA-seq, MIX cells became enlarged and spindle-like. Single-cell-derived clones also initially displayed dendritic melanocyte-like morphology, whereas late-stage SC1, SC3, and SC4 cultures exhibited enlarged and relatively flattened cell bodies with fewer prominent dendritic processes (Figure 1E). These observations suggested that lifespan extension was accompanied by cellular remodeling.

### 3.2. hTERT-Overexpressing Melanocytes Remained Closer to Primary Melanocytes than Melanoma Cell Lines at the Global Transcriptomic Level

**Figure 2:**
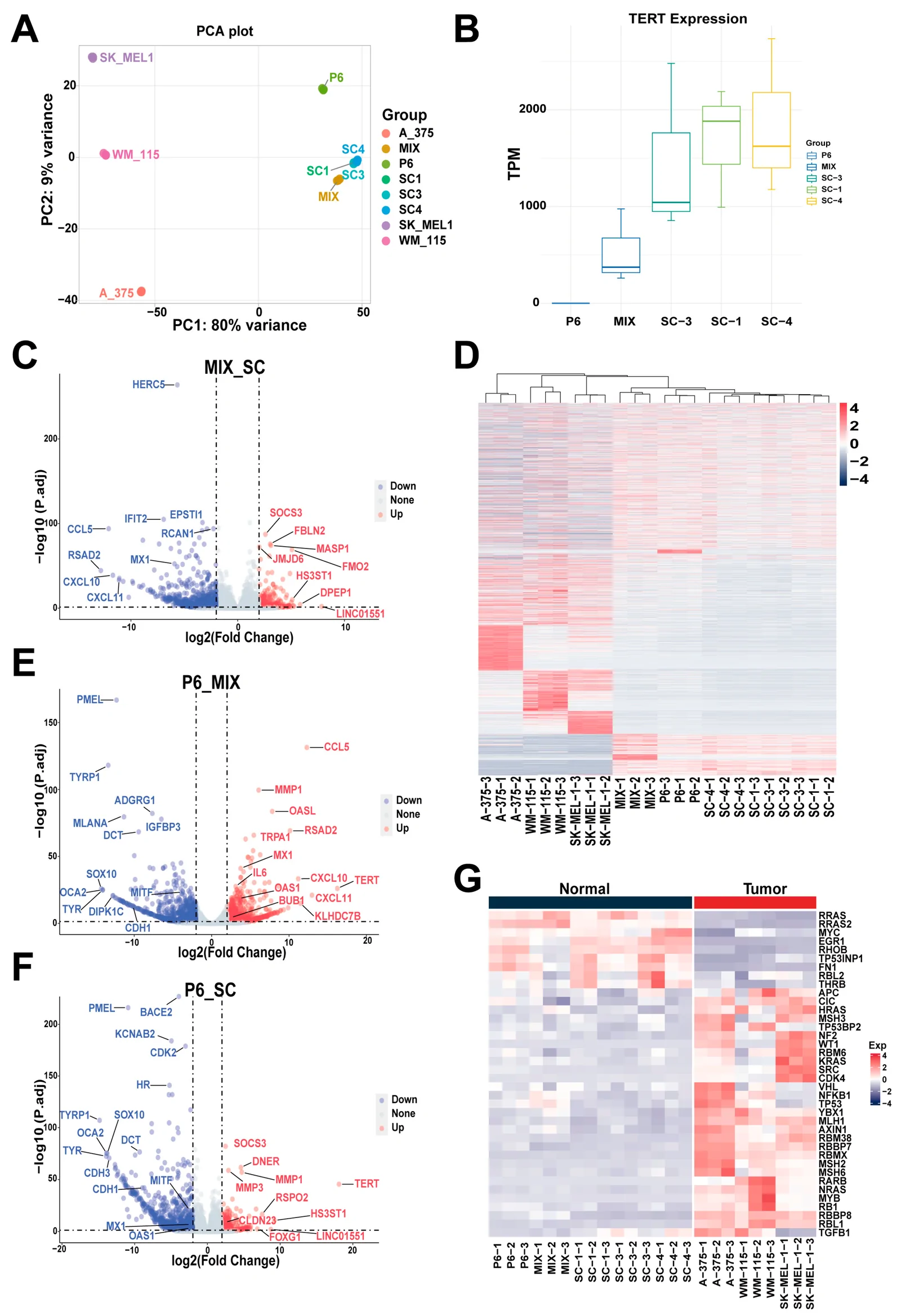
RNA-seq-based evaluation of hTERT-overexpressing melanocyte models. (**A**) PCA plot of P6, MIX, SC1, SC3, SC4, A375, WM-115, and SK-MEL-1 samples. (**B**) TERT expression in P6, MIX, and SC groups shown as TPM. (**C**) Volcano plot for SC versus MIX. (**D**) Hierarchical heatmap showing global gene-expression differences among all RNA-seq samples. (**E**) Volcano plot for MIX versus P6. (**F**) Volcano plot for SC versus P6. (**G**) Heatmap of curated tumor-related genes comparing non-tumor melanocyte model groups with melanoma cell lines.

To verify sustained hTERT overexpression and evaluate model fidelity, we performed RNA-seq on P6, MIX, SC1, SC3, SC4, A375, WM-115, and SK-MEL-1 cells. Principal component analysis (PCA) separated melanoma cell lines from primary and hTERT-overexpressing melanocyte groups, with PC1 explaining 80% of the variance (Figure 2A). The hTERT-overexpressing MIX and SC groups clustered closer to P6 cells than to the melanoma lines, whereas A375, WM-115, and SK-MEL-1 occupied distinct positions, consistent with substantial transcriptomic heterogeneity among melanoma models. TERT transcript levels were nearly absent in P6 cells, increased in MIX cells, and were highest in SC lines (Figure 2B). The elevated TERT expression in SC cultures may reflect clonal selection for cells with stronger hTERT expression and greater proliferative capacity during single-cell expansion.

Differential expression analysis showed extensive gene-expression changes during both hTERT overexpression and single-cell clonal expansion. Volcano plots for MIX versus P6, SC versus P6, and MIX versus SC highlighted marked upregulation of TERT and multiple stress-, immune-, extracellular matrix-, and proliferation-associated genes (Figure 2C,E,F), together with downregulation of melanocyte lineage and pigmentation-associated genes such as PMEL, TYRP1, MLANA, DCT, SOX10, and MITF in relevant comparisons (Supplementary Table S1).

Hierarchical clustering and heatmap analysis further supported the PCA results. MIX and SC transcriptomes resembled P6 melanocytes more closely than the melanoma comparators, whereas melanoma cell lines displayed divergent expression patterns (Figure 2D). These data indicate that hTERT-overexpressing epidermal melanocyte cultures provide a closer transcriptomic model of primary melanocytes than the tested melanoma cell lines. Because hTERT expression and immortalization can raise concerns about tumor-associated changes, we examined a curated set of tumor-related genes in P6, MIX, SC, and melanoma comparator cells. Tumor-related gene expression clearly separated the non-tumor melanocyte model groups from melanoma cell lines (Figure 2G). MIX and SC samples clustered with P6 rather than with A375, WM-115, or SK-MEL-1. These transcriptomic data do not demonstrate malignant transformation after lentiviral hTERT overexpression.

### 3.3. Gene Set Enrichment Analysis Linked Morphological Changes to Loss of Pigmentation, Adhesion, and Dendrite-Related Programs

**Figure 3:**
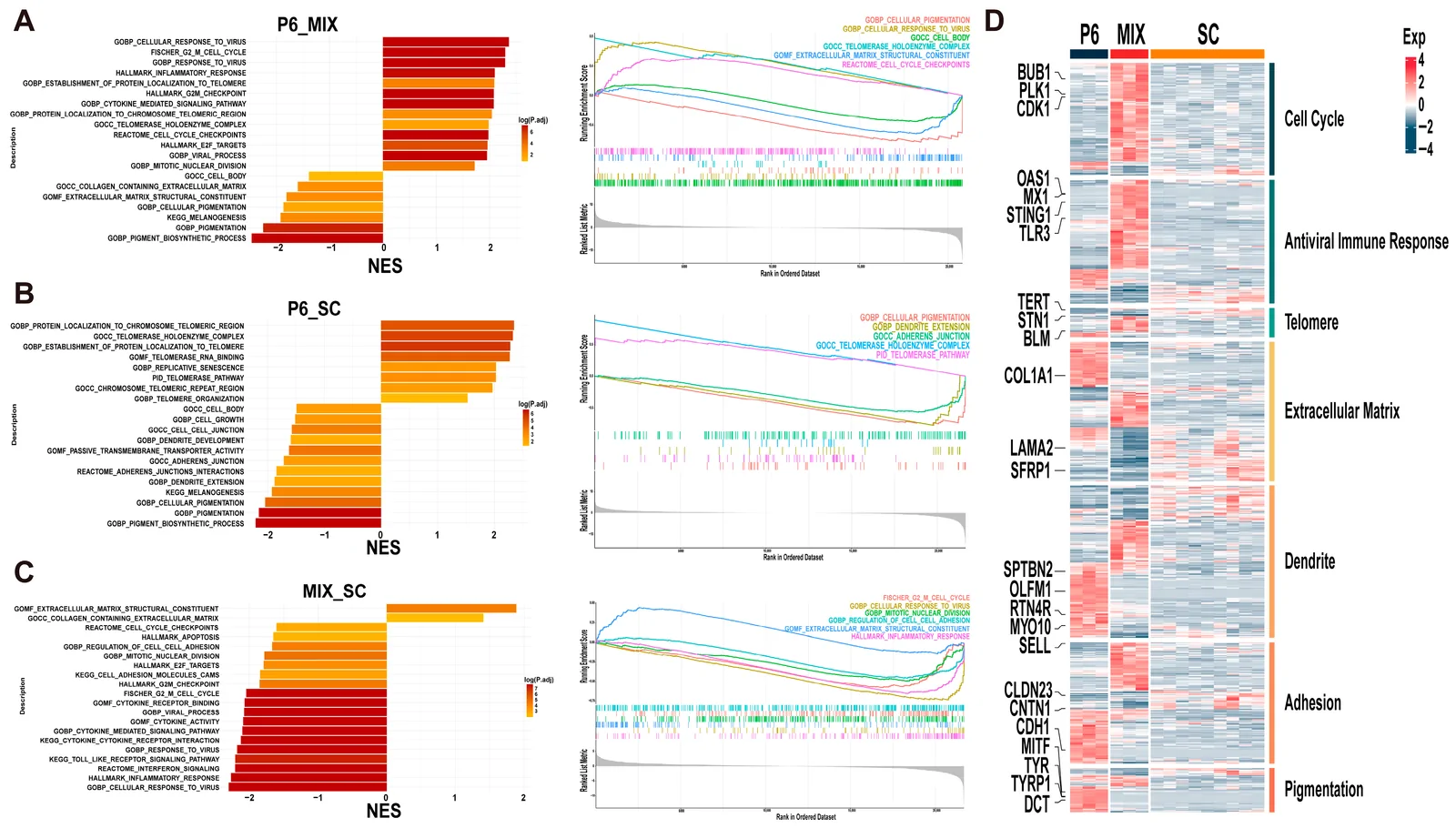
Functional remodeling during hTERT overexpression and clonal selection. (**A**) GSEA summary for MIX versus P6. (**B**) GSEA summary for SC versus P6. (**C**) GSEA summary for SC versus MIX. (**D**) Functional-module heatmap integrating representative genes related to cell cycle, antiviral immune response, telomere biology, extracellular matrix, dendrite-associated programs, adhesion, and pigmentation.

Gene set enrichment analysis provided a pathway-level view of functional remodeling. In MIX compared with P6 cells, antiviral immune response, telomerase activity, cell-cycle checkpoint, and nuclear division gene sets were enriched, whereas cell body, extracellular matrix, and melanin biosynthesis gene sets were reduced (Figure 3A). These changes are consistent with the observed shift from multipolar dendritic melanocyte morphology toward enlarged spindle-like cells after hTERT overexpression.

In SC compared with P6 cells, telomere-related gene sets were enriched, whereas cell body, cell growth, adhesion, dendrite-related, and pigmentation gene sets were reduced (Figure 3B). In SC compared with MIX cells, extracellular matrix-related programs were reactivated, whereas adhesion, cell-cycle checkpoint, and antiviral response programs were reduced (Figure 3C). These transcriptional changes were accompanied by the enlarged and less dendritic morphology observed in late-stage SC cultures.

A functional-module heatmap integrating representative genes from enriched pathways showed coordinated changes across seven biological domains: cell cycle, antiviral immune response, telomere biology, extracellular matrix, dendrite-related programs, adhesion, and pigmentation (Figure 3D). Together, these analyses demonstrate that lentiviral hTERT-mediated lifespan extension preserved global similarity to primary melanocytes but altered several functions that are central to melanocyte biology.

## 4. Discussion

This study established and evaluated hTERT-overexpressing primary human epidermal melanocyte models. The optimized lentiviral strategy substantially extended culture lifespan, and both MIX and SC cells maintained a global transcriptomic profile closer to primary melanocytes than to melanoma cell lines. These findings supported hTERT-overexpressing melanocytes as a useful alternative to melanoma cell lines for selected in vitro studies of epidermal melanocyte biology [1,2].

Previously established human melanocyte culture systems provide an important context for evaluating the present model. Normal human epidermal melanocytes can undergo substantial proliferation and serial propagation under optimized mitogenic conditions, including phorbol ester/cholera toxin-based systems [10,11], serum-free or low-serum media with defined growth factors [38,39], and keratinocyte-supported culture models that better preserve dendricity and melanization [40,41]. In contrast, genetically lifespan-extended melanocyte models using hTERT together with SV40 early-region genes [42] or hTERT/CDK4R24C/p53DD [43] provide more robust long-term expansion but introduce additional perturbations in cell-cycle and tumor-suppressor pathways. The present model uses lentiviral hTERT overexpression without deliberate disruption of p53/RB signaling and achieved more than 10 additional passages; however, our transcriptomic and morphological analyses indicate that this advantage is accompanied by biological drift, particularly after single-cell clonal selection. Thus, optimized primary cultures, keratinocyte-supported pigmentation models, and genetically lifespan-extended melanocytes should be regarded as complementary rather than interchangeable experimental systems, with model selection guided by the biological question being addressed.

From a practical perspective, these results suggest that hTERT-overexpressing melanocytes are best considered lifespan-extended melanocyte-derived models rather than direct substitutes for primary melanocytes. The MIX population may be preferable to single-cell-derived clones when preservation of the parental melanocyte state is important, because clonal cultures showed higher TERT expression and more pronounced reductions in pigmentation- and dendrite-associated programs. Accordingly, hTERT-extended melanocytes may be useful for experiments requiring repeated passaging, genetic manipulation, drug treatment, or transcriptomic analyses, provided that the biological phenotype relevant to the intended application is validated. For studies centered on melanogenesis, differentiation, adhesion, or normal melanocyte physiology, primary melanocytes or hTERT-extended cultures independently validated for these specific functions remain preferable.

The major caveat is that hTERT-mediated lifespan extension was not function-neutral. Morphological changes were evident in both the mixed population and single-cell-derived clones, and RNA-seq analysis revealed coordinated changes in pigmentation, adhesion, dendrite-associated pathways, extracellular matrix programs, telomere biology, antiviral immune response, and cell-cycle regulation. This pattern is biologically plausible. Mature melanocytes are highly differentiated, dendritic, and specialized for pigment production [44], whereas long-term culture and clonal outgrowth require survival and proliferation. The observed transcriptional changes may therefore reflect a combination of hTERT-mediated lifespan extension, altered proliferative state, long-term culture adaptation, and clonal selection rather than hTERT activity alone.

The pigmentation phenotype is particularly important for model selection. Melanin synthesis is a defining function of differentiated melanocytes [45,46], but pigmentation-associated genes and melanin biosynthesis pathways were downregulated in late hTERT-overexpressing cultures. Previous studies indicate that melanocyte proliferation, morphology, and pigmentation can change in a coordinated manner in response to environmental or culture-related stimuli. For example, UVB exposure has been reported to reduce melanocyte cell number and Ki-67 expression while increasing dendrite formation and, at specific doses, melanin content [47]. Therefore, the reduction in pigmentation-associated programs observed in our hTERT-extended cultures should not necessarily be interpreted as a direct effect of hTERT alone, but may also reflect changes in proliferative state and long-term culture adaptation. Accordingly, these cells might be suitable for experiments requiring expandable melanocyte-like cells, telomere manipulation, or broad melanocyte transcriptomic context, but they might be less suitable for studies focused on melanin production unless differentiation conditions are optimized.

Adhesion and extracellular matrix findings also require attention. Previous work has shown that hTERT can influence adhesion and migration independently of telomerase activity in some cellular contexts [26]. In the present epidermal melanocyte system, adhesion-related pathways were generally reduced after hTERT overexpression and clonal selection, whereas extracellular matrix programs changed dynamically between MIX and SC cells. These discrepancies across cell types suggest that hTERT-associated functional drift depends on the differentiation state and lineage biology of the parental cells.

The comparison with melanoma cell lines provided practical context. A375, WM-115, and SK-MEL-1 are widely used, but their transcriptional profiles differ substantially from each other and from non-tumor melanocytes [48,49]. MIX and SC cells remained closer to P6 cells than to these melanoma comparators, and tumor-related gene expression did not show a melanoma-like shift. However, this finding does not exclude subtle genomic instability or transformation potential. Future work should include karyotyping, long-term growth curves, senescence markers, soft agar assays, and in vivo tumorigenicity testing if these cells are to be distributed as stable model lines.

This study has additional limitations. First, the primary melanocyte source was a single donor-derived commercial culture, which limits generalizability across donor age, sex, ancestry and anatomical site. Second, the analysis focused on late-stage cultures; intermediate time points would help separate immediate lentiviral response, hTERT expression, culture adaptation, and clonal selection. Third, the current work used transcriptomic inference to assess function. Direct assays of melanin content, tyrosinase activity, dendrite morphology, adhesion, migration, and proliferation would strengthen the biological interpretation [50]. Fourth, melanocyte proliferation, morphology, differentiation, and pigmentation are strongly influenced by culture conditions and are biologically interrelated. Consequently, the present experimental design cannot fully distinguish direct hTERT-dependent effects from secondary consequences of altered proliferative state, prolonged culture adaptation, and single-cell clonal selection. The transcriptional and morphological changes observed here should therefore be interpreted as features associated with hTERT-mediated lifespan extension in this particular culture system rather than as effects attributable exclusively to hTERT itself. Future studies will therefore include independent donor-derived melanocytes, longitudinal analyses across passages, direct functional assays of melanogenesis and melanocyte differentiation, and genomic-stability and transformation-related assessments. These studies will help define appropriate passage windows and quality-control criteria for routine use of hTERT-extended melanocyte models.

## 5. Conclusions

Overall, lentiviral hTERT overexpression is a practical strategy for expanding epidermal melanocyte cultures, but the resulting cells should not be treated as simple replacements for primary melanocytes. hTERT-extended melanocytes should therefore be used cautiously in studies involving pigmentation, adhesion, differentiation, and normal melanocyte physiology. Future studies should explore strategies to extend melanocyte lifespan while preserving their characteristic cellular functions.

## Data Availability

The RNA-seq data have been deposited in Gene Expression Omnibus under accession number GSE338364.

## Supplementary Materials

The following supporting information can be downloaded at: https://www.mdpi.com/article/10.3390/biom16091239/s1, Table S1: Table_S1_All_DEGs.xlsx.

## Abbreviations

The following abbreviations are used in this manuscript:

CDS	coding sequence
DMEM	Dulbecco’s modified Eagle medium
FBS	fetal bovine serum
GEO	Gene Expression Omnibus
GO	Gene Ontology
GSEA	gene set enrichment analysis
hTERT	human telomerase reverse transcriptase
HPV	human papillomavirus
KEGG	Kyoto Encyclopedia of Genes and Genomes
MEM	minimum essential medium
MIX	mixed hTERT-overexpressing melanocyte population
MOI	multiplicity of infection
MSigDB	Molecular Signatures Database
P6	passage-6 primary melanocytes
PBS	phosphate-buffered saline
PCA	principal component analysis
RNA-seq	RNA sequencing
SC	single-cell-derived hTERT-overexpressing melanocyte clone
SV40	simian virus 40
TPM	transcripts per million
TU	transducing units
